# Crafting Inconspicuous Luxury Brands Through Brand Authenticity in China

**DOI:** 10.3389/fpsyg.2022.826890

**Published:** 2022-05-03

**Authors:** Zhiyan Wu

**Affiliations:** Department of Marketing, School of Management, Shanghai University of International Business and Economics, Shanghai, China

**Keywords:** inconspicuous branding, craft, brand authenticity, Chinese luxury branding, traditional culture

## Abstract

Currently, we are witnessing a trend toward subtle or absent hints of luxury, reflecting the rise of inconspicuousness. We seek to address why and how Chinese luxury brand managers, instead of matching conspicuous branding of many Western brands, develop inconspicuous strategies and craft authentic attributes in their brand communication. In the Chinese luxury brand context, we use the ethnographic research method with in-depth interviews, field visits, and photographs of eight Chinese luxury brands with inconspicuous preferences to reveal three main themes contributing to inconspicuousness. First, an inconspicuous approach of Chinese luxury brands is derived from the rise of inconspicuous consumption in China and a rejection of status brands due to being less famous than well-known Western brands, superficiality of status branding, and limited production capability. Second, we argue that inconspicuous branding can encompass developing luxury brands that avoid overtly displaying wealth and social status. Third, we identify three ways of crafting brand authenticity to build inconspicuous brands by using (a) nature to craft quality commitment dimension of authenticity (places and rare raw materials); (b) traditional Chinese craftsmanship and symbols to craft heritage dimension of authenticity; and (c) sincere stories (of how innovations are used in traditional craftsmanship), and the use of sustainability (sustainable raw materials, traditional craftsmanship, luxury production process, and saving resources) to craft sincerity dimension of authenticity in developing inconspicuous brands.

## Introduction

Luxury consumption in mainland China reached $9.83 billion in 2020, accounting for 80% of global luxury consumption (NPD.com, 2021). These figures demonstrate a promising future for the Chinese luxury brand market. However, much existing research examines how Chinese consumers conspicuously consume Western luxury brands and how such brands engage in status (conspicuous) branding in China (e.g., Bellezza et al., 2014; Kapferer, 2015; Mainolfi, 2020). For example, Kapferer (2015) indicates the inconspicuous branding in China because Chinese consumers love consume the large logo brands like LV to show their social status.

Research on luxury brands tends to focus on conspicuous or inconspicuous consumption, and few studies have explored how luxury brand managers inconspicuously build luxury brands. For example, Anaya (2013) recently pointed out that many traditional luxury brands were making their logos much more subtle or not visible at all. Inconspicuous brands encompass luxury brands but have low visual prominence, use quiet or discreet signals in their design, and avoid overtly displaying wealth and social status of consumers (Berger and Ward, 2010; Eckhardt et al., 2015; Wu and Luo, 2017; Pino et al., 2019). Such inconspicuous luxury branding methods offer differentiation from mainstream status branding (Han et al., 2010; Dion and Borraz, 2017) *via* culture (e.g., tradition or heritage) and performance (e.g., high quality).

Accordingly, Chinese luxury consumers have changed in recent years (Schroeder et al., 2015; Wu and Luo, 2017). For example, Wu and Luo (2017) observe a rise of inconspicuous consumption in China, identifying four types of inconspicuous consumers wishing to develop their identity. For instance, having explored Western products, some Chinese people return to their cultural origins and pursue a way of life based on those origins. In doing so, they become interested in subtler products with Chinese cultural elements—in particular, traditional cultural elements.

This change in Chinese consumers has prompted the emergence of Chinese luxury brands, including Shang Xia (which references traditional Chinese design and craftsmanship), Sand River (traditional Chinese cashmere design and craftsmanship), Ne•Tiger (traditional Chinese fur design and craftsmanship), Shiatzy Chen (traditional Chinese clothing design and craftsmanship), Shanghai Tang (reflecting Chinese clothing design and craftsmanship from the 1920s to the 1940s), and Franz (traditional Chinese porcelain design and craftsmanship).

Brand managers often develop crafted or authentic brands to differentiate them from others (e.g., Beverland, 2005; Campbell, 2005; Napoli et al., 2016). Campbell (2005) indicates that genuine craft branding involves helping consumers apply skill, knowledge, judgment, and passion through the experience of craft production and consumption while being motivated by a desire for self-expression. Furthermore, Beverland (2005) observes that brand managers typically use the production technique, history, tradition, and place to craft brand authenticity in the wine industry. His research further shows how wine brand managers craft authenticity to develop status brands. Moreover, Napoli et al. (2016) suggest that brand authenticity includes quality commitment, sincerity, and heritage dimensions.

Furthermore, in branding area, brand is driven by the development of technology (e.g., online platform, Meilhan, 2019), which also affect consumers’ perception and acquisition decisions (Drugău-Constantin, 2019; Graessley et al., 2019; Mirică (Dumitrescu), 2019; Birtus and Lăzăroiu, 2021; Rydell and Kucera, 2021; Vătămănescu et al., 2021; Watson and Popescu, 2021). Based on the theories of brand authenticity, inconspicuous consumption, this study reveals why and how luxury brand managers craft authenticity to create luxury brands with only a low visual prominence and use quiet or discreet signals in their design.

## Literature Review and Theoretical Background

### Luxury, Inconspicuous Consumption, and Inconspicuous Brands (Branding)

The concept of luxury traces to the Latin “luxuria,” which means “extras of life” (Danziger, 2005). Such unnecessary goods or services facilitate a luxurious lifestyle by providing indulgence or convenience that go beyond the essential minimums. Brand researchers often define by “luxury” the top categories of prestigious brands (Vigneron and Johnson, 2004). This term has recently evolved into “new luxury” or luxury for the masses, which involves affordability, mass-market spread, and emerging distinctions between social status and cultural capital (Eckhardt et al., 2015). In this new luxury world, there has been a surge in inconspicuous consumers who consume luxury products without publicly displaying their social status, and correspondingly, luxury brand managers are developing luxury brands inconspicuously.

Many researchers define inconspicuous consumption as that occurring when consumers do not publicly display their social status (e.g., Shove and Warde, 2002; Berger and Ward, 2010; Wu and Luo, 2017; see Table 1). Indeed, the phrase “inconspicuous consumption” used to refer to routine consumption of “ordinary” goods and services (Shove and Warde, 2002; Smith, 2007), but inconspicuous consumption now often intersects with luxury consumption (Sullivan and Gershuny, 2004; Berger and Ward, 2010). Keenly distinguishing terms, marketing researchers have mentioned that the concept of luxury should be redefined and separated from conspicuousness: inconspicuousness is the new conspicuousness (Eckhardt et al., 2015). The existing literature explores several types of inconspicuous consumption (Sullivan and Gershuny, 2004; Stacey, 2009; Berger and Ward, 2010; Belk, 2011; Wu and Luo, 2017). Berger and Ward (2010) identify a type of inconspicuous consumption in which consumers use subtle markers to distinguish themselves from the mainstream. Belk (2011) and Stacey (2009) describe a group of inconspicuous consumers who are rich and prefer not to provoke envy and anger in times of economic austerity. Sullivan and Gershuny (2004) point to a type of inconspicuous consumers who perceive luxury goods and services through the consumer’s imagined future use of purchases already made. Wu and Luo (2017) describe four types of inconspicuous consumers in China who construct and display three types of identity, which are also reflective of how consumers project these identities to themselves. Furthermore, a type of inconspicuous consumers who employ subtle signals is observable only to people with the requisite knowledge to decode their meaning (Wu and Luo, 2017; Makkar and Yap, 2018; Pino et al., 2019; Bellezza and Berger, 2020; Eckhardt and Bardhi, 2020). For example, Eckhardt and Bardhi (2020) investigate status signaling based on inconspicuousness and non-ownership while Bellezza and Berger (2020) reveal trickle-round signals, moving directly from the lower to the upper class, before diffusing to the middle class.

**Table 1:** Participants’ profiles.

Name	Gender/age	Background description	Interview location and date	Method of recruiting	Field visit
Mrs. Guo	Female/48	CEO of Sand River; master degree with 5 years of work experience in Germany; 26 years of work experience in this sector	Manufacturing site in Shanghai—9/2014	Recruited at a branding conference in Shanghai	Manufacturer visit
			Pasture site in Alxa—7/2020	Second-round interview	Pasture visit
Mr. Zhao	Male/34	Brand manager of Sand River; master degree with seven years of work experience in this sector	Manufacturing site in Shanghai—12/2020	Recruited *via* participants’ introduction	Same as Mrs. Guo’s
Mrs. Chen	Female/35	PR manager of Sand River; master degree with 8 years of work experience in this sector	Manufacturing site in Shanghai—9/2020	Recruited *via* participants’ introduction	Same as Mrs. Guo’s
Mr. Sun	Male/35	Brand manager of Shiatzy Chen; master degree with 7 years of work experience in this sector	Coffee shop in Beijing—9/2012	Recruited *via* participants’ introduction	/
Mrs. Yang	Female/30	Retail manager of Shiatzy Chen; BA degree with 8 years of work experience in this sector	Retail store in Shanghai —9/2012	Recruited from Shiatzy Chen’s retail store in Shanghai	Retail store visit
Mrs. Huang	Female/30	Retail manager of Shiatzy Chen; BA degree with 5 years of work experience in this sector	Retail store in Beijing—9/2013	Recruited from Shiatzy Chen’s retail store in Beijing	Retail store visit
Mrs. Zhang	Female/38	Brand manager of Shang Xia; master degree with 2 years of studying overseas; 12 years of work experience in this sector	Exhibition site in Shanghai—12/2012	Recruited *via* friends’ introduction	Exhibition site visit
Mrs. Liu	Female/33	Retail manager of Shang Xia; master degree with 1 year of studying overseas; 6 years of work experience in this sector	Retail store in Beijing—9/2015	Recruited from Shang Xia’s retail store in Beijing	Retail store visit
Mrs. Wu	Female/31	Retail manager of Shang Xia; master degree with 1 year of studying overseas; 5 years of work experience in this sector	Retail store in Shanghai -— 9/2017	Recruited from Shang Xia’s retail store in Shanghai	Retail store visit
Mr. Li	Male/35	Brand manager of Ne Tiger; BA degree with 9 years of work experience in this sector	Coffee shop in Beijing—9/2015	Recruited *via* participants’ introduction	/
Mrs. Wang	Female/32	Retail manager of Ne Tiger; BA degree with 5 years of work experience in this sector	Retail store in Beijing—9/2015	Recruited from Ne Tiger’s retail store in Beijing	Retail store visit
Mr. Zhang	Male/~42	CEO of Ne Tiger; BA degree with 20 years of work experience in this sector	Own office in Beijing—9/2015	Recruited *via* friends’ introduction	Office and design center visit
Mr. Shi	Male/42	Regional manager of Chow Tai Fook; master degree with 18 years of work experience in this sector	Own office in Wuhan—7/2015	Recruited *via* friends’ introduction	Office and design center visit
Mr. Fan	Male/43	Regional manager of Chow Tai Fook; master degree with 18 years of work experience in this sector	Own office in Liaoning—6/2015	Recruited *via* friends’ introduction	Office and design center visit
Mrs. Zhou	Female/35	Retail manager of Chow Tai Fook; BA degree with 10 years of work experience in this sector	Retail store in Shanghai—8/2020	Recruited from Chow Tai Fook’s retail store in Shanghai	Retail store visit
Mrs. Wu	Female/32	Retail manager of Qeelin; BA degree with 8 years of work experience in this sector	Retail store in Shanghai—8/2015	Recruited from Qeelin’s retail store in Shanghai	Retail store visit
Mrs. Xu	Female/36	Retail manager of Qeelin; BA degree with 12 years of work experience in this sector	Retail store in Shanghai—8/2015	Recruited from Qeelin’s retail store in Shanghai	Retail store visit
Mrs. Ma	Female/30	Retail manager of Qeelin; BA degree with 6 years of work experience in this sector	Retail store in Beijing—9/2015	Recruited from Qeelin’s retail store in Beijing	Retail store visit
Mr. Wu	Male/40	Regional manager of Franz; master degree with 15 years of work experience in this sector	Own office at Shanghai—7/2013	Recruited *via* participants’ introduction	Office and design center visit
Mrs. Zhu	Female/32	Retail manager of Franz; BA degree with 8 years of work experience in this sector	Retail store in Shanghai—7/2013	Recruited from Franz’s retail store in Shanghai	Retail store visit
Mrs. Xu	Female/33	Retail manager of Tangy Collection; BA degree with 9 years of work experience in this sector	Retail store in Beijing—7/2020	Recruited from Tangy Collection’s retail store in Beijing	Retail store visit

Stemming from this force accordingly, the rise of inconspicuous consumption leads to that of inconspicuous brands sending brand signals that are not readily apparent or visible to most consumers (Berger and Ward, 2010). Branding has been examined from a variety of perspectives (e.g., Fournier and Alvarez, 2019). Anaya (2013) recently pointed out that many traditional luxury brands are making their logos much more subtle or not visible at all. Eckhardt et al. (2015) suggest that subtle inconspicuous branding should become popular in the region as the market matures in China, suggesting that Shang Xia is subtle enough and emotionally resonant, has a great brand origin myth and is bringing back pride in Chinese craftsmanship; the company is creating local brands that will radiate luxury more subtly. Inconspicuous brands are conspicuously absent from the cited studies. However, researchers have mentioned the phenomenon of inconspicuous branding. Therefore, this paper examines how and why Chinese luxury brand managers develop Chinese luxury brands inconspicuously.

### Craft Consumption (Branding) and Authenticity (Branding)

The verb to “craft” refers to products or fashion created with skill, especially by hand (Morhart et al., 2015; Elliot, 2016). This kind of activity often includes weaving, hand block printing, embroidery, silversmithing, jeweler’s work, furniture-making, etc. The existing literature on craft consumption and brand development suggests that craftwork has tended to be viewed as romantic and associated with the sentiment of unease with the modern world and either yearning for a return to an earlier preindustrial age or nurturing unrealistic dreams of future postindustrial utopias (Campbell, 2005). Craft consumption is defined as activities in which consuming skillful, inalienable, humane, authentic, and creative work and typically needs skill, knowledge, judgment, and passion while being motivated by a desire for self-expression (Campbell, 2005; Elliot, 2016). Such craft consumption has been identified as typically encountered in the case of, e.g., some luxury goods marketed as authentic.

The term authenticity is normally defined as genuineness, reality or truth of something, sincerity, innocence, originality, being natural, honest, simple, and unspun (Beverland and Farrelly, 2010). Here, authenticity is original and pure with the objective ideal of authenticity. Correspondingly, authentic objects cannot contain alterations contrary to history, quality, or art and cannot represent dilution, as the granting authority is being the natural and functional (e.g., someone who has had plastic surgery is inauthentic because she or he is not original or pure and has gone against nature and history). In this way, the brand authenticity construct is multifaceted and built around perception of heritage (including nostalgia and cultural symbolism), sincerity, and quality commitment (including craftsmanship and design consistency, Napoli et al., 2016). In each perceptive concept, consumers choose specific hints to redefine authenticity according to the notions of time, place, culture, self, and others. However, authenticity is sometimes subjective, such as authenticity as forms of harmony, balance, or delight (Beverland et al., 2008). Thus, researchers also examine authenticity as being fabricated, iconic, indexical, hypothetical, and rising to the point of hyperauthenticity (Schallehn et al., 2014; Fritz et al., 2017).

In each case, consumers with different levels of cultural capital seek different hints to signal authenticity (Holt, 2002), and connotations of authenticity shift over time (Brown et al., 2013). For example, Brown et al. (2013) suggest that the style of Titanic Movie is authentic for some because they are mythic. When linking authenticity to time and place, consumers assert tradition (Napoli et al., 2016), which might be a key facet of consumers with a low cultural capital (Peterson and Anand, 2004).

Authenticity is of great importance for strong brands because it creates a key element of a crucial brand identity (Kapferer, 2015). Many brand managers are using brand histories and historical and traditional elements as sources of market value and a “cultural marker of legitimacy and authenticity” (Brown et al., 2013, p. 19). Thus, this paper examines why and how Chinese luxury brand managers craft authentic brands inconspicuously.

Our research goal at the outset was to identify the dominant structure of the managerial motives of Chinese luxury brand managers (Eckhardt et al., 2015). The emergent structures we identify show that inconspicuous brands can be analyzed through authenticity formation based on managerial practices. Our participants used narratives of Chinese luxury brands with managerial motives and authenticity formation. These narratives portray managerial motives in the context of authenticity through interaction with nature, Chinese heritage, and Chinese traditions. By using narratives to ground the meanings of Chinese luxury brands, we balance the emphasis on inconspicuous brands within brand strategy studies of the structuring of brand manager motives against the existing status branding strategy. Our study offers a complementary extension by revealing the influence of heritage and traditions on Chinese luxury brand managers’ meanings.

## Materials and Methods

Arnould and Wallendorf (1994) suggested that the data collection of marketing-oriented ethnographical study involved field observation (e.g., field visits, photographs, and interviews). This ethnographically inspired study draws from field visits, interviews, and photographs over an 8-year period. In contrast to the common status branding strategy, based on the theories of brand authenticity and inconspicuous consumption, this study reveals why and how luxury brand managers and owners create inconspicuous brands through crafting brand authenticity. Using purposive and opportunistic sampling (Patton, 2014), potential participants (store managers) were approached in Chinese luxury retail stores. A snowball sampling approach was then used, which involved asking participants to introduce other potential participants (Patton, 2014). Bearden et al. (1993) further stated that multiple methods of data collection were often employed in ethnographic research to access different domain of experience that deviate from each other. The data collection process of this research included retail visits, manufacturing site visits, exhibition visits, and field visits and a set of interviews that took place from 2012 to 2020. This article focuses on the interview data and incorporates insights derived from other elements of the study, including photographs and news releases.

Interviews took place at firms’ locations, exhibition sites, and retail stores in Shanghai, Beijing, Wuhan, Liaoning, and Alxa and lasted from 75 to 150 min. In total, we interviewed 21 CEOs, brand managers, regional managers, and retail store managers. Before interview, consent forms had been signed by participants regarding the use of their data. These interviewees consisted of seven males and 14 females ranging in age from 30 to 48 years (see Table 2). The number of participants in this research was 21, which was suitable for ethnographic study because Wallendorf and Arnould (1991) suggested that generally speaking, the number of participants in ethnographic study is often more than 10. After interview, I asked participants whether they allow to appear their first name in this research and all participants agreed to do that.

**Table 2:** Profiles of Chinese luxury brands considered in this paper.

Brand name	Year of brand establishment	Founder and art director/gender	Logo visibility	Brand mission
Shang Xia	2008	Jiang Qionger/female	Invisible	Inherit and nurture traditional Chinese craftsmanship
Sand River	2004	Guo Xiuling/female	Invisible	Promoting traditional cashmere craftsmanship
Shiatzy Chen	1978	Wang Chen Caixia/female	Invisible	Promoting traditional Chinese embroidery-making craftsmanship
Ne Tiger	1982	Zhang Zhifeng	Invisible	Promoting China’s national dress with traditional Chinese craftsmanship of Kesi (tapestry), Yunjin (brocade), embroidery, and paper cutting
Chow Tai Fook	1929	Zheng Yudong/male	Invisible	Promoting traditional Chinese gold-casting craftsmanship
Qeelin	2004	Dennis Chan and Guillaume Brochard/male	Invisible	Promoting traditional Chinese jewelry making craftsmanship
Franz	2001	Franz Chen/male	Invisible	Promoting traditional Chinese china-making craftsmanship
Tangy Collection	2008	Liang Zi/female	Invisible	Promoting traditional Chinese gambiered silk-making craftsmanship

In 2012 and 2013, we met and interviewed six managers, and two of them were contacted through friends’ (participants) introductions. Another four participants were recruited from field visits where we imitated buying their companies’ products, met retail managers at Shiatzy Chen and Franz’s retail stores in Shanghai and Beijing, and asked if they would be willing to be interviewed. For example, during a field visit when we imitated buying Shang Xia’s product, we met retail store managers of Shiatzy Chen and asked if one of them would be willing to participate in an interview. She accepted our request and introduced us to the company’s brand manager.

In 2014 and 2015, we met and interviewed 10 managers; four of them were contacted through friends’ (participants) introductions, and we met one of them at a brand conference and asked her to accept our interview. Another four participants were recruited from field visits, where we imitated buying their companies’ products, met retail managers at Ne Tiger, Qeelin, and Shang Xia’s retail stores in Shanghai and Beijing and asked if they would be willing to be interviewed.

In 2017 and 2020, we met and interviewed six managers; two of them were contacted through participants’ introductions, and one of them was conducting a second-round interview in the Alxa area of Inner Mongolia at a cashmere production farm, where she invited the interviewer for a visit. Another three participants were recruited from field visits, where we imitated buying their companies’ products, met retail managers at Shang Xia, Chow Tai Fook, and Tangy Collection’s retail stores in Shanghai and Beijing, and asked if they would be willing to be interviewed.

Among the potential participants, many love to craft their brands inconspicuously, while a small number of Chinese luxury brand owners and managers love to develop Chinese brands conspicuously. We did not focus on Chinese luxury brand owners and managers who developed status brands and conducted interviews with a group of participants who loved to craft brand authenticity inconspicuously. Moreover, we are not trying to link all status branding to Western goods. The informants varied in terms of gender, age, educational background, and level of involvement with luxury brand development, and we established the brand profile based on what was mentioned during the interviews (see Table 2).

Arnould and Wallendorf (1994, p11) indicated that “rather than a truly separable stage in a sequence, the process of interpretation building typically begins in the filed with a set of field note entries, including records of revelatory incidents and other working hypotheses or ideas about recurrent patterns.” In this study, participants answered questions about the branding strategies they adopted and why and how they carried out their branding strategies. We took notes both in the fields and during the interviews. The analysis began in the first year with summaries of findings organized by type of data, namely, interviews and photographs. Based on the patterns in the data, we then established preliminary branding categories. Subsequently, because we became familiar with Chinese luxury branding methods and noted repetitions in participants’ responses, we shifted our attention to producing and evaluating themes and cultural principles. We derived themes from the substantive content of data across categories with respect to luxury branding and authenticity construction. One experienced researcher interpreted all the interview data. Our analysis entailed triangulation across data sources (interviews, field visits, and photographed artifacts), with particular attention to comparing the Chinese luxury branding methods identified by managers and brand owners, their understanding of the design motifs and the potential related meanings, and their descriptions of how and why they conducted inconspicuous branding through crafting authenticity. To increase the credibility or trustworthiness of this ethnographic interpretation, we employed repetition and variation data, as suggested by Arnould and Wallendorf (1994).

## Findings

**Figure 1:**
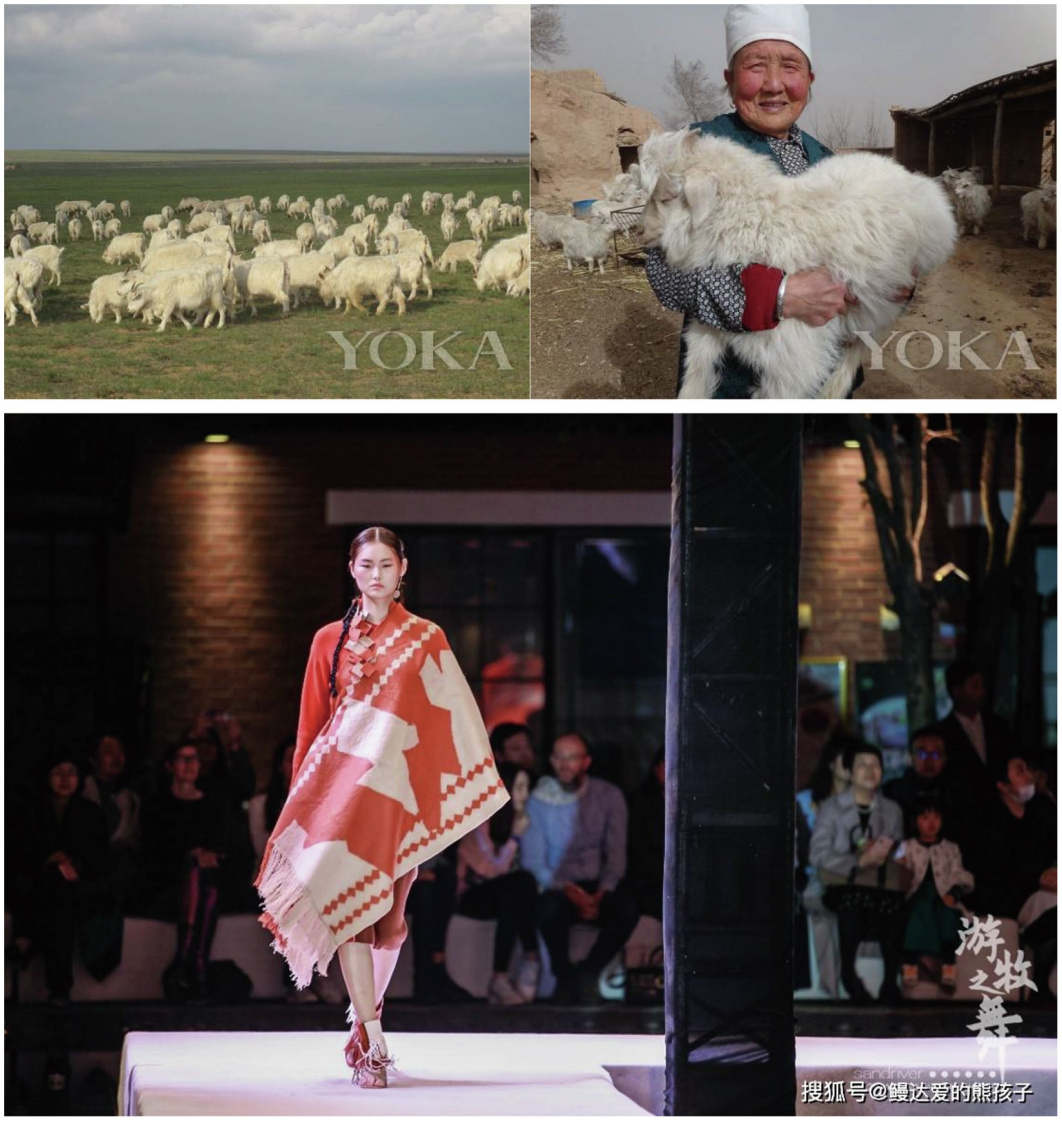
Findings. The three photos displayed were all taken and kindly provided by Sand River’s CEO Mrs. Guo.

First, inconspicuous brands encompass luxury brands but have low visual prominence, use quiet or discreet signals in their design, and avoid overtly displaying wealth and social status of consumers (Berger and Ward, 2010; Wu and Luo, 2017). Crafting brand authenticity includes quality commitment (including craftmanship), heritages (including nostalgia), and sincerity dimensions (Napoli et al., 2016). Our analysis is based on the theories of brand authenticity and inconspicuous consumption. Figure 1 shows our results.

### Inconspicuous Branding as a Strategy

#### Rise of Inconspicuous Consumers in China

Berger and Ward (2010) describe a group of inconspicuous consumers who avoid products with clear brand identifiers. These consumers reject ostentatious status symbols or feel guilty about being conspicuous consumers (Belk, 2011). Correspondingly, Wu and Luo (2017) demonstrate the rise of inconspicuous consumption in China and identify four types of inconspicuous consumers. Postrel (2008) demonstrates that conspicuous consumption of nouveau-rich consumers in BRIC (Brazil, Russia, India, and China) nations is shifting from being conspicuous to inconspicuous as these nations become wealthier. For example, Mrs. Jiang, the founder and art director of Shang Xia, said in public that (qq.com, 2018),

“--- after having widely experienced Western brands, some Chinese are returning to their cultural origins and pursuing a way of life based on those origins. They come to be interested in subtler products, which do not have superficial Chinese elements (e.g., the dragon) but have authentic traditional Chinese craftsmanship and its values ---. Our brand conforms to this trend. ----The Chinese millennials are defining their Chinese identity by using Chinese wisdom in their ways of life, in their consumption---”

Mrs. Guo, the CEO of Sand River, agrees with Mrs. Jiang’s and further states that

“Not only do middle-aged Chinese consumers love to consume Chinese brands, but some young Chinese consumers are also loving Chinese products now. They love Chinese products that have good design and quality. They do not like to consume ostentatious status symbols of well-known Western luxury brands, which are thought of as superficial consumption. They love exquisite products with cultural meanings. The majority of Sand River’s consumers are Chinese. They truly love the cashmere craftsmanship of Sand River. Certainly, they love Sand River’s crafting of traditional Chinese culture and esthetics. For example, this summer we cooperated with Forbidden Palace Cultural Gems to design two cashmere scarves with unique palace elements. Our Chinese consumers love them so much! They are becoming the most popular items in our catalog---”

Wu and Luo (2017) indicate that a type of inconspicuous Chinese consumers appreciates esthetic and functional elements of Chinese brands instead of ostentatious status symbols of well-known Western luxury brands. Statements of both Mrs. Jiang and Mrs. Guo confirm the findings of Wu and Luo (2017). They further demonstrate that Chinese luxury consumers are shifting from being conspicuous to inconspicuous by consuming well-performing products. Furthermore, Chinese consumers enjoy buying products with excellent traditional Chinese craftsmanship and choose brands that meet such consumers’ needs. Thus, the rise of inconspicuous consumption has facilitated the development of inconspicuous Chinese brands, such as Shang Xia, Sand River, Ne Tiger, Shiatzy Chen, Sand River, Franz, Qeelin, Chow Tai Fook, and Tangy Collection.

#### Rejection of Status Brand Positioning

##### Being Less Famous Than Well-Known Western Brands and Superficiality of Status Branding

Humphreys and Carpenter (2018) indicate that some American wine brands are not only innovative compared to top high-tech brands (e.g., Apple, Google, Amazon, and Facebook) but can also lead the market with cultural elements that influence consumer preferences. Accordingly, Chinese luxury brands have a short history and are less well known than Western ones (e.g., LV or Chanel) but use inconspicuous branding as their strategy to meet inconspicuous consumers. For example, Mrs. Guo, the CEO of Sand River, suggests that

“Our brand does not focus on logos. We do not pay attention to consumers who want to buy brands to show their status. Our brand is a new brand. We could not compare to famous Western brands. We do not like to show status. I think it is superficial. We just offer our consumers a meaningful brand that represents a life attitude: love fine goods, love life.”

All participants demonstrate their agreement with Mrs. Guo’s statement. For example, Mr. Sun, the brand manager of Shiatzy Chen, further explains that

“You know, Western luxury brands, such as LV, Chanel, and Fendi, have a longer history than ours. They are also more famous than us. Such a weakness of ours could not be dealt with in a short period of time. For example, our marketing fees are certainly lower than theirs (Western famous brands’). So, we found a new way to brand ourselves, that is, inconspicuous branding ---”

The above statements typically show that all managers and CEOs recognize the reality that Chinese luxury brands are less well known than famous Western brands. The polled individuals further suggest that their brand missions are to guide consumers to enjoy fine goods and love life, and strongly reject status branding because they think that it is superficial. Moreover, Mr. Sun’s statements imply that Chinese luxury brands have to engage in inconspicuous branding to compensate for the low-fame weakness of their brands.

##### Limited Production Volume of Rare Raw Materials and Craftsmanship

Status brands require many rare materials to support their large consumption volume. Goulding and Derbaix (2019) suggest the use of material to develop brand authenticity. However, Mrs. Chen, the public relations manager of Sand River, Mrs. Wang, the retail manager of Ne Tiger, and Mrs. Yang, the retail manager of Shang Xia all suggest that their brands cannot adopt a status branding strategy. For example, Mrs. Chen, the public relations manager of Sand River, indicates that

“The annual production volume of our cashmere is limited, so we cannot promote our brand as a status brand. We make great efforts to improve our product quality and traditional craftsmanship to attract the consumers that truly know and love us, rather than consumers who blindly follow status branding”.

Mr. Sun, the brand manager of Shiatzy Chen, Mrs. Wang, the retail manager of Ne Tiger, and Mrs. Liu, the retail manager of Shang Xia all agree with Mr. Chen’s statements. Mrs. Wang, the retail manager of Ne Tiger, further indicates that

“---we promote our brands with an invisible logo. We use the traditional Chinese craftsmanship of Kesi (also called *k’o-ssu*, Chinese cut silk tapestries and the essence of traditional Chinese silk art) to produce Huafu (Chinese national dress). It takes more than 30 days to produce one Huafu with Kesi craftsmanship. So, production volumes of some of our products are limited.”

Mrs. Liu, the retail manager of Shang Xia, moreover explains that

“Although we are a new brand, we are very famous in this sector because our brand is supported by Hermes. However, our products have invisible logos, as do Hermes’. We pursue a peaceful lifestyle with a good taste. It takes us a long time to find the cherished traditional Chinese craftsmanship. We are nurturing the craftsmen’s culture, which requires a long time. Moreover, the annual production volume of some raw materials in our products is very limited. For example, Zitan (red sandalwood) is used in our furniture, but some red sandalwood has a limited volume of production because in 500 years only 1cm of red sandalwood can grow.”

These three descriptions all demonstrate that the companies reject being status brands because it is snobbish, and the production volumes of their raw materials and the scale of craftsmanship are limited. Furthermore, the companies love to promote their branding inconspicuously because they have the responsibility for facilitating a peaceful life with a good taste. Furthermore, Mrs. Liu’s description indicates that Shang Xia is a well-known brand, but its brand mission is to inherit and promote Chinese craftsmanship culture. Shang Xia chooses to not be a status brand and to instead be an inconspicuous brand.

### Crafting Authenticity to Build Inconspicuous Brands

Authenticity is often real (Napoli et al., 2016). Firms demonstrate authenticity by strategically establishing their identity in the marketplace, claiming themselves to be small craft producers that use (among other things) time-honored techniques and natural ingredients as a means of competitive differentiation (Carroll and Swaminathan, 2000). Napoli et al. (2016) further indicate that authentic value also arises from quality commitment. In China, many luxury brand owners (e.g., Shang Xia and Sand River) make every product as a craft without an obvious logo. They promote the use of rare materials from special places to skillfully and innovatively craft products by hand to show their quality commitment rather than status branding.

#### Use of Nature to Craft Quality Commitment

Napoli et al. (2016) suggest that brand authenticity includes quality commitment. Carroll and Swaminathan (2000) demonstrate that authentic value strategically arises at firms that claim themselves to be small craft producers that use (among other things) time-honored ways and natural ingredients (e.g., places and rare raw materials) as a means of competitive differentiation. We argue that the use of nature to craft quality commitment dimension of brand authenticity in developing inconspicuous luxury brands. The managers and CEOs of Chinese luxury brands state that they use nature to craft brand authenticity inconspicuously.

##### Use of Places to Craft Quality Commitment Authenticity

Authenticity is reflected in relating the brand to a particular place to show quality commitment (Beverland, 2005). Six brands of my cases use places as points of reference, which was mentioned in the interviews, evidenced in one field visit and, among the secondary sources we consulted, appeared in relation to eight cases (see Table 3 for details). This is explained by the realization that many of the firms simply faced limitations on what they could produce and thus needed a form of competitive differentiation. The relationship to a place reinforced a unique aspect, granting authenticity to the product. However, many Chinese luxury brand owners have to temper their commitment to authenticity as purity (expression of place) with the need to ensure consistency of quality and evoke emotions in consumers.

**Table 3:** Use of places and raw materials to create authenticity, as summarized from interviews.

Brand name	Place	Rare, top-end raw material
Shang Xia	E.g., traditional bamboo weaving craftsmanship in southwest China’s Sichuan province	E.g., Zitan, Hetian jade, and Mongolian cashmere
Sand River	Traditional cashmere craftsmanship in the Alxa region of inner Mongolia	Cashmere from Alxa
Shiatzy Chen	E.g., traditional Su embroidery craftsmanship in the Jiangsu province	/
Ne Tiger	E.g., traditional Chinese craftsmanship of Kesi (tapestry) in the Jiangsu province	E.g., material made of furs from China’s northeast
Chow Tai Fook	/	/
Qeelin	/	/
Franz	Traditional Chinese china-making craftsmanship in the Jingde town of the Jiangxi province	/
Tangy Collection	Traditional Chinese gambiered silk-making craftsmanship in Shunde district, Foshan city, Guangdong province	Gambiered silk

This view of authenticity was expressed in the commitment to *terroir*, with products differentiating themselves due to terroir differences being regarded as “real.” For example, the following quote demonstrates this viewpoint, relating it to a sense of uniqueness and a defined positioning. This is the outward appearance of marketing.

**Figure 2:**
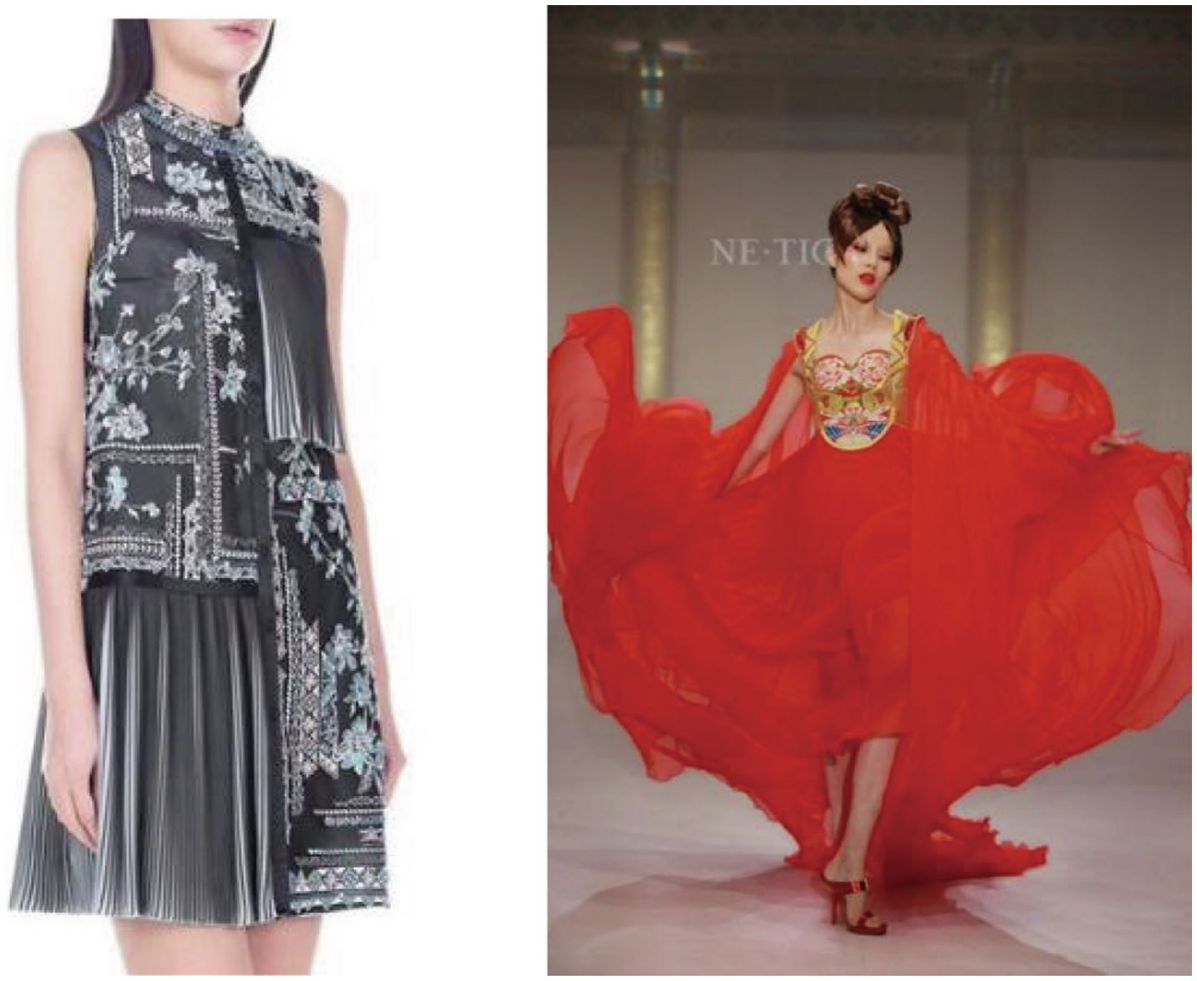
Alxa’s pasture, goats and herdsmen, and the best clothes made from the discarded cashmere. The photo displayed on the left was taken and kindly provided by Shiatzy Chen’s brand manager Mr. Sun. The photo displayed on the right was taken and kindly provided by Ne Tiger’s retail manager Mrs. Wang.

“The question of our cashmere being a luxury product is attributed to us having a centuries-long track record of perfect cashmere production, and yes, we are at the top end of the cashmere market. Ours is of high quality, and the resulting prestige of our cashmere was due at first to a very typical of Alxa of Inner Mongolia –albeit very real – concept of ‘terroir’, the meaning of which, as you surely know, is a combination of soil, microclimate, lamb and grass varieties, etc. ---The Alxa desert area is very dry throughout the year. This area is suitable for this kind of grass, which grows easily in salt marshes, such as pearl grass, red sand grass, Alternanthera philoxeroides, etc.; this type of grass has high salinity. Alxa’s white cashmere goats that eat this kind of grass will have a high degree of lean meat but not fat. In this way, Alxa’s white cashmere goats eat this kind of grass and produce fine cashmere” (Mrs. Guo, the CEO of Sand River; 48; see Figure 2).

Mr. Zhang, the CEO of Ne Tiger, made a similar statement. He said that Ne Tiger fur’s raw materials came from the world’s top four fur auction houses. These high-end raw materials positioned Ne Tiger as a luxury brand in the fur industry.

Descriptions by Mrs. Guo and Mr. Zhang suggest that their companies use the *terroir* as a positioning statement and a guiding philosophy in several ways to thus create and assure authenticity of this aspect (see Figure 2).

##### Use of a Rare and Top-End Raw Material to Craft Quality Commitment Authenticity

Authenticity is reflected in relating the brand to a particular material to show quality commitment (Beverland, 2005). We argue that the linking of a brand to a place or the use of a particular material leads inconspicuous brand managers to craft quality commitment authenticity *via* the material’s scarcity, high quality, or being good for health. In four of my cases, commitment to the use of a scarce, high-quality, and healthy raw material was mentioned in the interviews, with evidence described in nine interviews and eight field visits, and among the secondary sources from related news releases, it appeared in relation to eight cases (see Table 3 for details). For example, Mrs. Zhang, brand manager of Shang Xia, agrees with Mr. Zhao’s statement and further indicates that

“We often use high-quality and rare materials (e.g., Zitan, Hetian jade, and Mongolian cashmere) to produce our products. For example, our Zitan furniture is very popular among our consumers, and we often use Zitan to produce furniture. *Zitan* is an extremely dense wood that sinks in water. The fine texture of wood grain is especially stable and difficult to crack, which is suitable for intricate carving. The Zitan tree is relatively rare and grows quite slowly because the tree requires 800 years to become wood. It is also called a wood emperor, and was widely used in ancient palaces, particularly in the Ming dynasty. It is also good for health and can freshen the mind, sedate, nourish the spleen, the stomach and the liver, and produce other health effects.”

Mrs. Jiang, the founder and art director of Shang Xia, describes the company as follows:

“In 2014, Christie's Auction House held a special sale of 20 Shang Xia limited edition treasures, noting that the pieces resonated with modernity, and featured a sense of design, craftsmanship and rare raw materials. It was the first time an auction was held for Chinese artwork with such contemporary design, and all 20 pieces were sold, with some fetching a handsome amount” (Chen, 2018; see Figure 2).

Mrs. Jiang, the founder and art director of Shang Xia, backed by the French luxury company Hermes, spoke as follows at the celebration ceremony for the permanent collection of Shang Xia’s Xi Pi lacquer at the British Museum:

“I want to start from the original source of living art to seek the true meaning of craftsmanship in contemporary life.”

Mrs. Zhang simply explains the hard, firm, healthy, and scarce attributes of Zitan wood and demonstrates the impression of authenticity made by a rare raw material. Such material is one of the prominent attributes within Shang Xia, which for the first time was featured in an auction held for Chinese artwork and achieved sound results. Furthermore, the expressions “the original source” and “the true meaning” by Mrs. Jiang refer to the use of a rare raw material to create authentic attributes of Shang Xia. All of the above narratives show that Sand River and Shang Xia craft brand authenticity by using scarce raw materials.

#### Use of Heritages to Craft Authenticity

Some brands create authenticity by using natural ingredients. However, Napoli et al. (2016) indicate that authentic value also comes from heritage (including tradition). In China, many luxury brand owners and managers (e.g., those of Shang Xia, Sand River, Ne Tiger, and Shiatzy Chen) craft authenticity through traditional Chinese craftsmanship.

##### Use of Traditional Chinese Craftsmanship to Craft Heritage Authenticity

**Figure 3:**
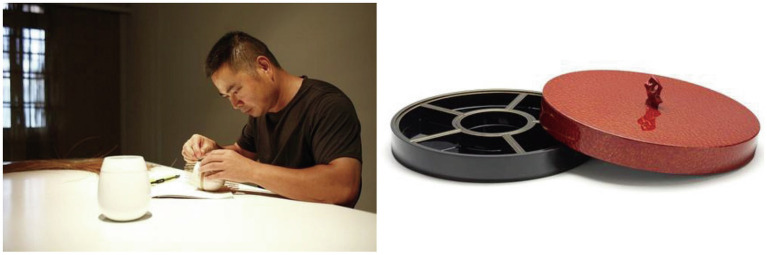
Shiatzy Chen’s Su embroidery and Ne Tier’s kesi craftsmanship in Huafu. The two photos displayed here were taken and kindly provided by Shangxia’s retail manager Mrs. Liu.

The linking of a brand to a place and a material or the use of traditional craftsmanship in production lead inconspicuous brand owners and managers to seek protection for the use of that name and traditional expressions associated with the production of luxury goods in the relevant areas. In eight of our cases, commitments to traditional craftsmanship in production were mentioned in the interviews, with evidence presented in 21 interviews and 18 field visits, and, among the secondary sources we consulted, appearing in relation to eight brands (see Table 2 for details). For example, Mr. Sun, one of brand managers of Shiatzy Chen, states that (see Figure 3).

“We often use traditional Chinese embroidery craftsmanship to make our products unique, cultural and natural. For example, in the summer of 2016, we used Su embroidery in our products. You know that Su embroidery is known for its delicacy and elegance. We invited a famous craftsman to work on our products, who is the inheritor of the intangible heritage of Su embroidery. When starting embroidery, one hand of the craftsman is on the frame, and the other is under it to create the embroidery. One piece of work takes her at least 60 days with more than 300,000 stitches needed to make the work (product) perfect in form, color and quality. This craftsmanship requires more time, energy and artistic ability to make the embroidery’s face smooth, the edge of the pattern neat and tidy, the stitches fine and closing without traces, and the color elegant and bright.”

Mr. Sun’s expressions “unique, cultural and natural” and “the inheritor of the intangible heritage of Su embroidery,” and the detailed description of how the Su embroidery is made all demonstrate that Shiatzy Chen uses Chinese embroidery to develop its brands.

**Figure 4:**
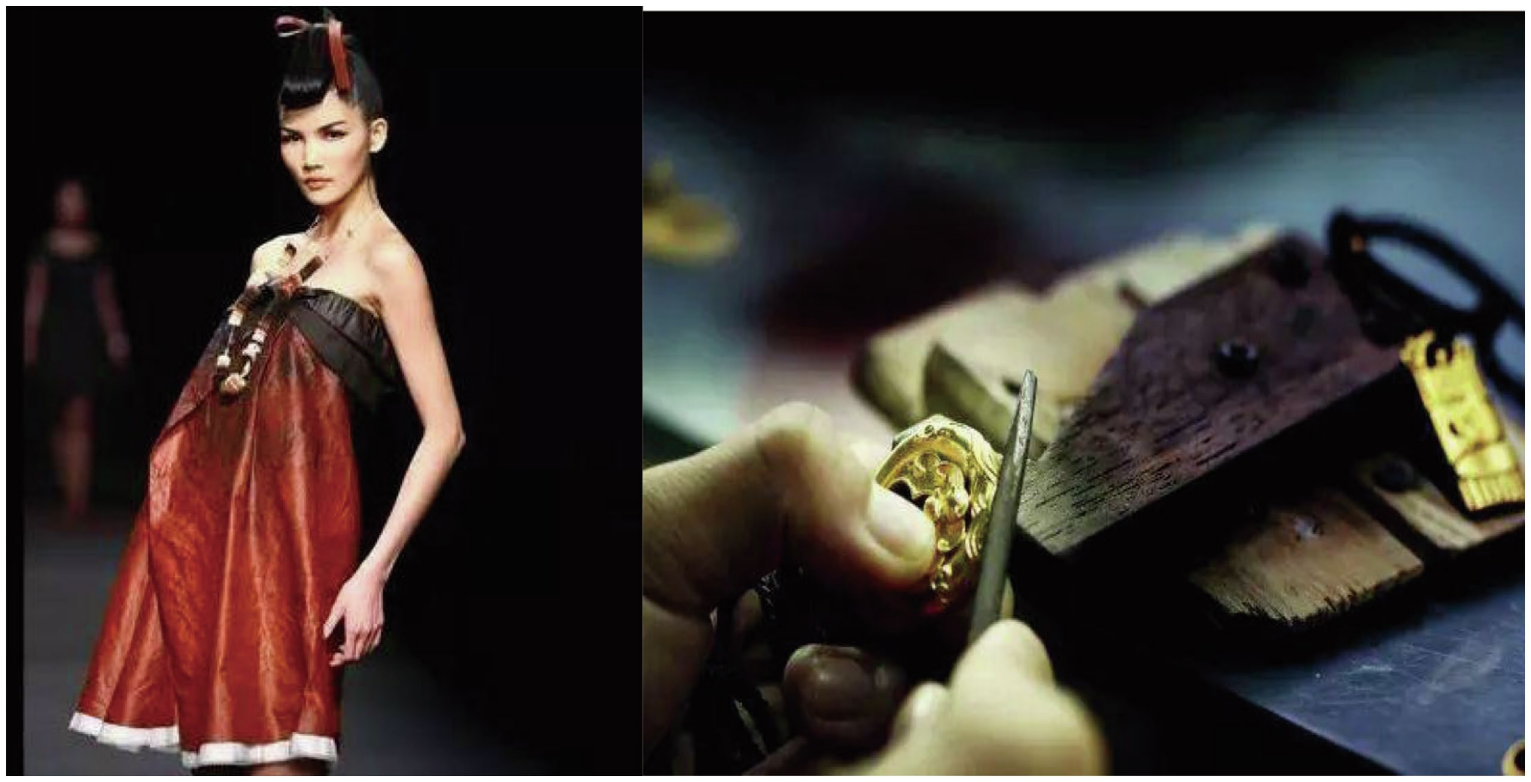
Craftsmanship of Shang Xia’s bamboo weaving teaware and Xi Pi lacquer collected by British Museum. The photo displayed on the left was taken and kindly provided by Tangy Collection’s retail manager Mrs. Xu. The photo on the right was taken and kindly provided by Chou Tai Fook’s regional manager Mr. Fan.

Mrs. Jiang spoke as follows at the celebration ceremony for the permanent collection of Shang Xia’s Xi Pi lacquer at the British Museum (see Figure 4):

“It not only represents the recognition and support of the world's top art venue for Chinese contemporary design and craftsmanship but also is a motivation for the cause we are following. ---I want to start from the original source of living art to seek the true meaning of craftsmanship in contemporary life.”

Mrs. Jiang’s words assert Shang Xia’s focus on crafting authenticity by the use of traditional Chinese craftsmanship. Discussing craftsmanship, Mr. Li, brand manager of Ne Tiger, described the brand as follows (see Figure 3):

“Our brand often uses traditional Chinese craftsmanship to develop our pieces. Huafu is our key product category. It is also called ‘China’s national dress,’ representing the essence of China’s culture and history. All of our Huafu pieces are made of the finest silk fabrics with ancient precious handicrafts, such as Kesi (tapestry), Yunjin (brocade), embroidery, paper cutting, etc. For example, Kesi, also called *k’o-ssu*, is Chinese cut silk tapestry and the essence of traditional Chinese silk art. Kesi adopts a weaving method that passes through the warp and breaks the weft, which makes the work look like having been carved and engraved and has a rich double-sided three-dimensional effect. Since the Song and Yuan Dynasties, Kesi has been extensively used to produce clothes for royal families, such as emperors and empresses.”

The words of “ancient precious handicrafts, such as Kesi (tapestry)---,” suggest the use of historical Chinese craftmanship in Ne Tiger’s inconspicuous brand development. The words of “since the Song and Yuan Dynasties” and “royal families” reveal the feeling which is revered, worshiped, cherished, and rare, and then develop the company’s brand authenticity.

Mr. Li, brand manager of Ne Tiger, further indicated that

“---Kesi is often called “an inch of Kesi and an inch of gold” and “the Holy Grail of Weaving” due to its extremely complex and meticulous weaving process and its rich and graceful features. Kesi craftmanship requires passing through the warp and breaking the weft, which enables the work to look like having been carved and engraved and then produces a rich double-sided three-dimensional effect. Because of the extremely mature and reliable Kesi craftsmanship in the Tang dynasty, craftsmen at this stage are able to use a variety of valuable materials, such as pure gold and silver threads and peacock feathers, to weave Kesi. It usually takes three years to learn the basic skills of Kesi, while making a good item usually takes over 20 years of apprenticeship and practice.”

The vivid descriptions show the high added value within Kesi work, the complex and unachievable features within Kesi craftmanship, the degree of difficulty to learn this craftmanship. Such descriptions make Ne Tiger worshiped, precious and cultural, and then create the impression of authenticity.

##### Use of Traditional Chinese Symbols to Craft Heritage Authenticity

Napoli et al. (2016) that the use of cultural symbols and traditions can develop the heritage dimensions of brand authenticity. Authenticity judgments may be formed from iconic cues, and the extent to which an object or event is a reasonable reconstruction of the past (Grayson and Martinec, 2004). In the context of China, all luxury brand owners and managers interviewed for this study state that they often use sincere storytelling to show how they craft traditions in their branding. For example, Mrs. Zhang, brand manager of Shang Xia, continues to describe the brand as follows (see Figure 4):

“The body of a Xi Pi lacquer box is crafted from black wood with red-spotting Xi Pi lacquer craftsmanship. Such craftsmanship is a traditional Chinese lacquer technique. In this box, the red-spotting Xi Pi lacquer is on the surface, and the refined black polished lacquer is inside, which creates a rippled pattern that embodies floating clouds or running water and signifies being ever-changing and never predictable. Furthermore, the shape of this box is sparkled by a traditional Chinese zan plate. Zan refers to a "gathering" in traditional Chinese, and a zan plate puts together several small plates and develops a round flower shape symbolizing happiness and completeness.”

Mrs. Jiang, the founder and art director of Shang Xia, reinterprets her signature adaptability and states that

“In Chinese, Shang Xia means "ups and downs,” and “Shang" signifies "into the sky," representing the passage of history, craftsmanship and tradition, while "Xia" is "the earth," representing the future, new technology and a new material. The words “tradition(al)”, “history”, “embodies”, “signifies”, and “symbolizing’ indicate how Shang Xia uses traditional symbols to craft its brand authenticity through sincere stories” (Chen, 2018).

Furthermore, Mr. Sun, brand manager of Ne Tiger, continues to describe the brand as follows (see Figure 3):

“---This Huafu’s design (Huafu refers to China’s National Dress) is inspired by traditional Chinese symbols, such as phoenix, peony, red color, golden threads, etc. The phoenix is traditionally often used for imperial families, and the peony is the national flower in the Chinese tradition. We also use red cloth to make this item and golden threads to weave it. Both phoenix and peony with golden threads in a red cloth denote the Chinese aesthetics of distinguishing and graceful features ---”

The symbolic meanings of a phoenix, peony, red color, and golden threads in the company’s design express Ne Tiger’s affirmation of the use of traditional Chinese symbols to reveal its authenticity through sincere storytelling.

#### Use of Sincere Story of Innovation in Chinese Craftsmanship to Craft Sincerity

An effect of authenticity developed through a sincere story shows a creative blend of industrial and rhetorical attributes (Napoli et al., 2016). Beverland’s (2005) study of fine wine producers identified that the development of a sincere story consisting of demonstrable references to place, tradition, and non-commercial values was crucial to conveying brand authenticity. The sincerity that creates authenticity was attained through a demonstration of a relationship to a place, a rare material, handcrafted techniques, a famous craftsman, esthetics, culture, and a unique passion for innovative production, innovation, and the use of modern marketing techniques.

Napoli et al. (2016) further indicate that brand authenticity includes quality commitment, heritage, and sincerity dimensions. Sincerity dimensions are measured by sticking to its principles of demonstrating references to place, tradition, and non-commercial values. For example, Mrs. Zhang, a brand manager of Shang Xia, states that

“Our brand mainly uses craftsmanship exhibitions, retail space, official website communications and media publication to deliver sincere stories to our consumers. We seldom use extremely commercial methods to make our products. We often exhibit crafted products and invite famous craftsmen to demonstrate this kind of craftsmanship. Such exhibitions sincerely tell the stories of how famous craftsmen use innovative blends of endangered craftsmanship and modern techniques to meticulously create our products. Our purpose is not only to enable consumers to find out about our products but also to learn about our traditional craftsmanship. Such exhibitions are usually announced through news, videos, official websites and salesperson introductions.”

Mrs. Zhang’s manifesto demonstrates how Shang Xia applies sincere stories to create an impression of authenticity *via* craftsmanship exhibitions, retail space, official website communications, and media publications. Using publications in the media to develop sincere stories, Mrs. Jiang told China Daily how Shang Xia sincerely made traditional Chinese craftsmanship functional and modern to create authenticity (see Figure 4):

“Some of the country's traditional know-how has become solely for decoration, but has no function. For example, when I met a craftsman doing bamboo weaving in southwest China's Sichuan province, they were making an elephant for an entry into the Guinness Book of World Records. --- It is amazing, but it is not going to be used in your daily life. --- My team worked with the same Sichuan craftsmen to weave a ‘coat’ to cover a white porcelain tea set. The thin bamboo strips were softly woven, following the shape of the teaware and seamlessly melding one material into the other. In terms of functionality, the design has enhanced the safety of serving hot tea, but when people hold the cup, they will feel handmade craftsmanship.”

Mrs. Jiang’s statement suggests how Shang Xia sincerely tells the story of its innovation to make traditional Chinese craftsmanship functional *via* media publications (e.g., in China Daily). Mrs. Jiang further asserts that the best way of preserving China’s rich heritage and craftsmanship is with innovation, saying the following:

"For them, this was to record history instead of just caring about the finished work itself," --- "In my opinion, all the museums in cooperation with us are actually looking for, collecting and recording a microcosm of the highest level of life, techniques, artisanship, science and technology, art and design of a certain period in society" (Chen, 2018).

Mrs. Zhang states that Shang Xia uses sincere stories to develop its authenticity *via* craftsmanship exhibitions, retail space, official website communications, and media publications. Mrs. Jiang’s description suggests that Shang Xia’s sincere story is developed through sincerely making traditional Chinese craftsmanship functional and modern.

#### Use of Sustainability to Craft Sincerity

##### Making Raw Materials Sustainable

Amos et al. (2019) state that customer service perceptions emerge as a significant boundary condition for the perceived authenticity of sustainability efforts. Karaosman et al. (2020) suggest that sustainability in luxury often includes craftsmanship, which is nearly disappearing from sustainable development, protecting the environment, animal welfare, and raw materials, and improving employees’ working environment, gender equality, etc. Karaosman et al. (2020) further propose that when carrying out suitability programs, luxury firms should make raw materials, craftsmanship, and environmental protection more sustainable. When suitability programs are implemented at the product level, the focus on raw materials results in more market benefits for consumer value-added than does the focus on design initiatives (Karaosman et al., 2020). Our participants’ descriptions confirm the suggestion of Karaosman et al. (2020). For example, Mrs. Guo, the CEO of Sand River, explains that (see Figure 3):

“In 2016, we carried out a sustainable development strategy for our firm. This year, our firm only makes a profit, but we would like to make a large heartfelt investment in this sustainable development program. The reasons behind this are that I was born in this area and grew up here. I know how hard the life of herdsmen is. How cherished cashmere is! (She is excited with tears in her eyes) --- First, to make the cashmere industry engage in sustainable development, a fund was set up to help herdsmen establish family farms and special farms. We sent professional vets to help herdsmen check the health of their goats every month. We help them set up data on their goats’ health and growth conditions. Second, this fund was also to help the children of herdsmen obtain better education. For example, the Sand River’s literature and art scholarship for students of herdsmen who attend primary school has been offered for four years. We encourage them to accept art and literature education, nurture their art ambitions and enable them to express love for their hometown and craftsmanship.”

**Figure 5:**
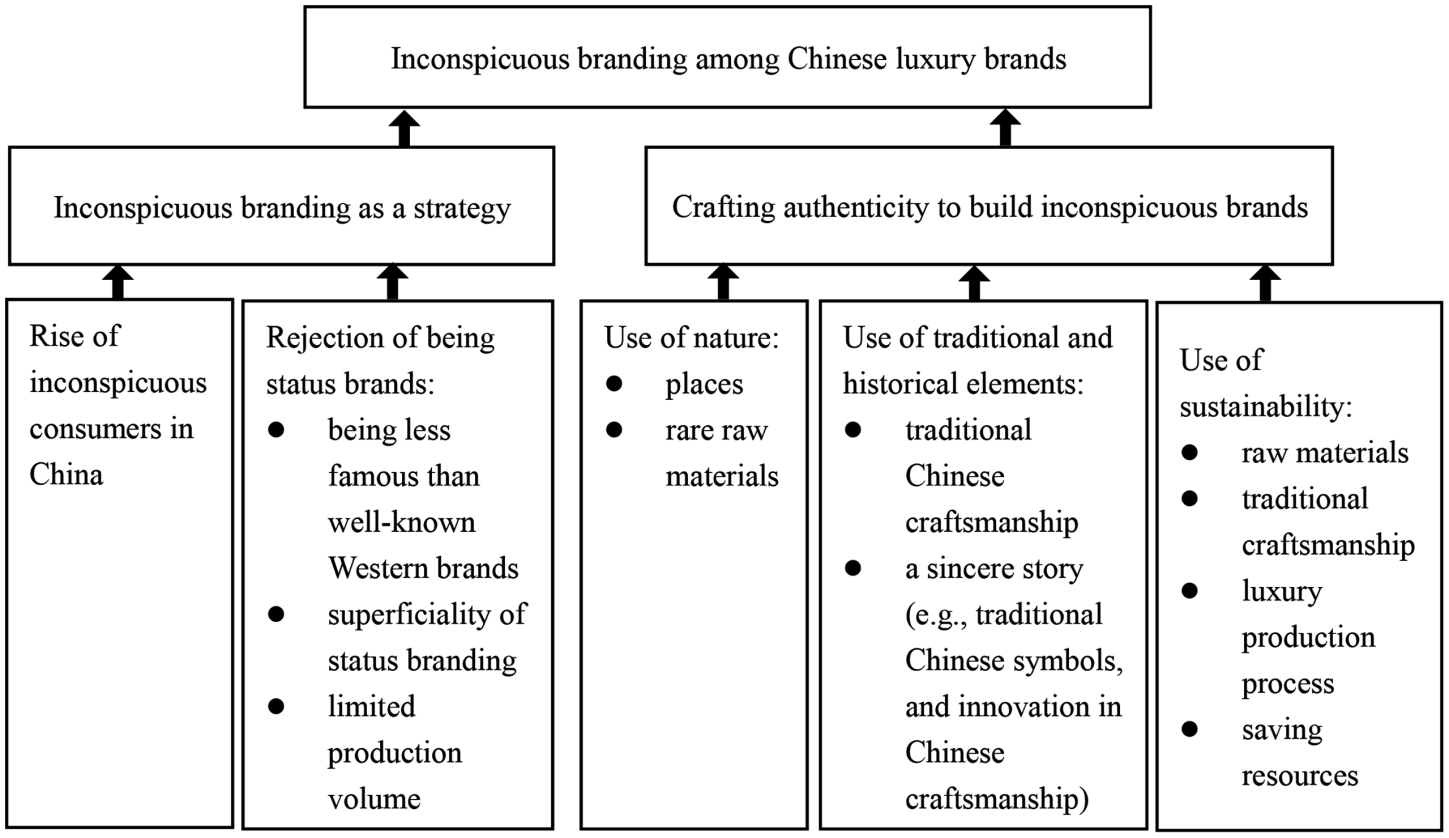
Tangy Collection and Chou Tai Fook’s gold-casting craftsmanship.

Mrs. Xu, a retail manager of Tangy Collection, agrees with the description by Mrs. Guo and further points out that (see Figure 5):

“We developed Tangy Silk, a new fabric, in 1995. It was developed based on gambiered silk and its traditional plant-based dyeing process. It is a top-quality and all-natural silk. It derives its unique colors and patterns from the extract of wild dioscorea cirrhosa and a 30-step process that involves plant-based dyeing, sun-drying and coating the fabric with unpolluted river mud. Gambiered silk appeared 1,000 years ago in China, but we invested much to give gambiered silk a new life. We genuinely love this fabric. My first view of this fabric with complex and eco-friendly craftsmanship filled me with awe. We devoted ourselves to conducting research on sustainable silk development. Last year, we were invited to show our all-natural and eco-friendly fabric in the sustainable and eco-friendly section of our Paris Fashion Show.”

The words “heartfelt,” “genuinely,” “love,” and “awe,” a sustainable development strategy and sustainable development all demonstrate that Sand River and Tangy Collection made great efforts to create authentic perceptions of sustainable development efforts. Specifically, Mrs. Guo’s words expound that Sand River develops raw materials sustainably by helping herdsmen improve the scientific skills of raising goats and improving the education of herdsmen’s children. Mrs. Xu’s words illustrate that Tangy Collection makes the raw material of gambiered silk and performs its craftsmanship while engaging in sustainable development.

##### Making Traditional Craftsmanship Sustainable

Autio et al. (2013) suggest that local food is associated with craftsmanship and artisan production and that consumers value it more sustainably and authentically. However, the participants’ narratives in this research show that brand owners and managers invest great efforts in making traditional craftsmanship sustainable to develop the impression of authenticity. For example, Mrs. Jiang, the founder and art director of Shang Xia, told Sohu.com (2019) that

“The reason Europe has become a place full of luxury brands is that Europe has a perfect craftsmanship industry chain, and then a rise in design ideology could be achieved at the top quality and taste level. However, in China, we have top-level traditional craftsmanship, but craftsmen are scattered among the folk and do not form an industry chain, and some techniques and skills are endangered or dying. So, Shang Xia is trying its best to develop the complete industry chain of craftsmanship. In doing so, we have established an R&D team to dig out craftsmanship and the related workshops. We learn the craftsmanship we have found and design new products based on that craftsmanship. We also invite university professors of literature and history and museum experts to provide craftsmanship inspiration and clues. The best way of protecting craftsmanship is not by giving money to craftsmen but by giving a market to them. Only when traditional craftsmanship is developed into products that are sold sustainably, we can enable traditional craftsmanship as a part of modern life, protect craftsmen, and improve the working environment of craftsmen.”

Mrs. Xu, a retail manager of Tangy Collection, confirmed Mrs. Jiang’s statement and further explained that

“We enable endangered gambiered silk, an all-natural and eco-friendly craftsmanship, to have a new life. We try our best to make it appear in the fashion and luxury industry, and then the increasing number of consumers know and consume it. This is the best way to protect the traditional craftsmanship of gambiered silk through sustainable development.”

The words “trying their best” and “making great efforts” show that Shang Xia, Tangy Collection, and their counterparts develop impressions of authenticity by making traditional craftsmanship sustainable. Furthermore, these narratives suggest how inconspicuous Chinese brand owners and managers use traditional craftsmanship to engage in sustainable development. Specifically, Mrs. Jiang demonstrates how Shang Xia’s R&D team makes great efforts and invests much in developing an integrated craftsmanship industry chain and selling its products to make such craftsmanship sustainable.

##### Making Luxury Production Processes Environmentally Friendly

Karaosman et al. (2020) suggest that when implementing sustainability at the production process level, environmental protection is a key aspect of making production processes sustainable. All our participants confirmed this suggestion of Karaosman et al. (2020) For example, Mrs. Guo, the CEO of Sand River, further indicates that

“To be honest, we think protecting the environment is our job! To prevent the problem of printing and dyeing chemicals polluting the environment and damaging health, we have engaged in the development of plant-dyed products to enable sustainable utilization of natural resources. Although this plant-dyed method costs us more, we think it is justified. For example, the isatis root printing and dyeing method is often used in our crafts. Isatis is a plant that has the function of removing heat and toxic materials. Therefore, the printing and dyeing method is environmentally friendly, and the clothes made using the isatis root printing and dyeing method are good for health.”

Mrs. Wu, a retail manager of Shang Xia, agrees with Mrs. Guo and further explains that

“We are sincerely committed to making our production environmentally friendly. For example, we use black sandalwood fruit as a dyeing material and make silk glossy, similarly to the gloss of lacquer and leather. We use this dyed silk to design a series of clothes named “the deep sense of lacquer.” The shine of such clothes resembles leather, but they are very soft, similarly to a baby’s skin. Many luxury firms use a chemical dying method to make clothes look shiny, but such methods are not environmentally friendly. So, we used a plant dyeing method and black sandalwood fruit to make the clothing shiny. The clothes made with the use of black sandalwood fruit as dyestuff have the function of soothing the nerves and facilitate good sleep because the fruit has this function.”

Mrs. Xu, a retail manager of Tangy Collection, confirmed descriptions by Mrs. Guo and Mrs. Wu, and further stated that

“We are proud that the material, the dyestuff and the process of making gambiered silk are all environmentally friendly. Here is our process of producing gambiered silk. First, we should squeeze Dioscorea cirrhosa and boil silk in Dioscorea cirrhosa’s juice. Second, we sprinkle silk with that juice and seal silk in that juice. Third, we dry silk under a gentle sunlight and daub the silk with sludge. Fourth, we soften silk in a fog and wash silk. Fifth, we obtain the finished product of gambiered silk.”

The words “honest, committed and proud” show that the mentioned inconspicuous brands create the perception of authenticity through their endeavors to make their production environmentally friendly. The brands use the plant printing and dyeing method with the sunlight drying technique to prevent pollution. Furthermore, surprisingly, their products that use plants for printing and dyeing are good for health.

##### Saving Resources

Karaosman et al. (2020) further suggest that when sustainability programs are implemented at the production level, the focus on saving resources results in more market benefits for consumer value-added than does the focus on environmental concerns. All our participants confirm this finding of Karaosman et al. (2020). For example, Mrs. Guo, the CEO of Sand River, states that

“From the bottom of my heart, I think saving resources is more important than environment concerns. This does not refer to the fact that environmental friendliness is not a way to make products sustainable and authentic, to which end the government has strict policies to prevent pollution and many luxury firms can do so. However, not all luxury firms have the capability to save every resource. For example, we held a cashmere Haute Couture fashion show with the title of “zero discarded material.” We always commit to making the luxury industry engage in sustainable development and call upon luxury firms to pay more attention to environmental protection, saving resources, and reusing the discarded material. In this show, for instance, the earrings and headwear that the model wore were all made from discarded cashmere. In particular, the last yet the best ready-made clothes in this show were made from the discarded cashmere too!”

Mr. Fan, a regional manager of Chow Tai Fook, agrees with Mrs. Guo’s statement and further indicates that (see Figure 5):

“We use electronic receipts instead of paper receipts to reduce paper waste. We carried out this program in May 2018. By financial year 2020, we saved 1,154 sheets of size A5. This program is very popular among our colleagues and consumers. We also try our best to save resources such as electricity and water. For example, at our production and retail sites, we try our best to use a natural lighting environment design and LED energy-saving lamps. We also try our best to use materials fully ---!”

The statements of both Mrs. Guo and Mr. Fan demonstrate that making a great effort to save resources could allow these luxury firms to develop the impression of authenticity. The expressions “from the bottom of my heart” and “try our best” all demonstrate the firms’ great endeavors to make production sustainable and create a sense of authenticity.

## Discussion

For nearly a decade, marketers have witnessed a decoupling of conspicuousness and luxury, as the signaling ability of conspicuous luxury goods has been diluted. Many elite consumers and far-seeing marketers are turning away from traditional logo-emblazoned luxury goods in favor of more discreet inconspicuous luxuries (Eckhardt et al., 2015). Eckhardt et al. (2015) offers the concept and trend of inconspicuous consumption, but they do not offer why and how to develop inconspicuous brands. This paper provides a type of inconspicuous branding through craft brand authenticity to explored the inconspicuous brand strategy of Chinese luxury brands, defined as marketing luxury products without overtly displaying wealth and social status. In contrast to status branding of Western luxury brands (e.g., Dion and Borraz, 2017), Chinese luxury brand managers and CEOs think that status brands are very shallow and want to develop meaningful luxury brands even though they have weaknesses in developing status brands, such as having less famous brands than Western counterparts and a limited production capability to support status branding. This contributes to a situation in which if Chinese luxury brands become famous, they still develop themselves inconspicuously.

Based on the three dimensions of developing brand authenticity by Napoli et al. (2016), we have also identified three ways of crafting brand authenticity inconspicuously for Chinese luxury brands. However, Napoli et al. (2016) suggest quality commitment dimension of brand authenticity, but they do not mention the use of nature to develop quality commitment to develop brand authenticity. Beverland (2005) suggests the use of a place to craft brand authenticity in the wine industry. However, our findings include the use of not only places to craft brand authenticity inconspicuously in the Chinese luxury branding context but also rare raw materials.

Furthermore, Napoli et al. (2016) indicate that authentic value arises from heritage. Based on the theory of Napoli et al. (2016), our findings demonstrate that the authentic value of inconspicuous luxury brands arises from traditional Chinese craftsmanship and tradition Chinese symbols. Vivid and in-depth descriptions by 21 participants reveal that the cherished traditional Chinese craftmanship and the traditional symbols are most effectively used to create an authentic impression of inconspicuous Chinese brands in the luxury industry.

Third, Beverland (2005) identified that the development of a sincere story consisting of demonstrable references to place, tradition, and non-commercial values was crucial to conveying brand authenticity. We argue that the sincere descriptions of how innovations are used in traditional heritage products show authentic value within inconspicuous Chinese luxury brands.

Fourth, Napoli et al. (2016) do not mention the use of sustainability to develop sincerity. Amos et al. (2019) suggest that significantly higher authenticity is attained as a result of sustainability efforts in above-average than in below-average service quality contexts. The research of Amos et al. (2019) does not show how to use sustainability to create authenticity but shows that higher service quality leads to greater authenticity of sustainability efforts. Our findings are the first to manifest the sincerity dimension of authentic value that arises from saving resources and using sustainable raw materials, traditional craftsmanship, and luxury production processes. Sustainability is a very important topic in the luxury industry. We often think that this topic is typically embraced by famous Western luxury brands. However, Chinese luxury brand managers and owners can also genuinely implement this strategy and therefore create impressions of authenticity inconspicuously.

Our findings help redefine constructs in the paradoxical situation of inconspicuous branding of luxury brands, adding a layer of complexity to the inconspicuous branding concept. Research on status (conspicuous) branding has shown that brand managers and owners apparently use readily visible and large logos to allow consumers to display wealth and social status (e.g., Dion and Borraz, 2017). Further studies might focus on the context of inconspicuous brand development and attempt to distinguish attraction to visual elements, such as bright colors or striking designs, from social psychology aspects, such as using a brand to indicate one’s identity and brand association. From a managerial perspective, these insights into the form of inconspicuous branding may help brand managers better understand how to manage brands “inconspicuously.”

## Conclusion and Contributions

Building upon the recent research on inconspicuous consumption and authentic branding, this study employs ethnographic study with 21 interviews and four pictures to reveal the practices of inconspicuous brand managers and CEOs and consumers in China (see Figure 1). We argue that inconspicuous branding can encompass developing luxury brands that do not enable consumers to overtly display wealth and social status. We identify three ways of crafting brand authenticity to build inconspicuous Chinese luxury brands: (1) the use of nature to craft quality commitment authenticity (places and rare raw materials); (2) the use of traditional Chinese craftsmanship and symbols to craft heritage authenticity; and (3) the use of sincere stories (of how innovations are used in traditional craftsmanship), and the use of sustainability (sustainable raw materials, traditional craftsmanship, luxury production process, and saving resources) to craft sincerity authenticity in developing inconspicuous brands.

Our research has two contributions. First, our findings help advance understanding of inconspicuous consumption and its impacts. Inconspicuous luxury branding in China may suggest a societal turn away from the concerns of communicating wealth and privilege to a preference for subtle signals and tastes. We offer a type of inconspicuous branding through crafting brand authenticity by crafting brand authenticity. Beverland (2005) discusses crafting authenticity for status brands, this research examines why and how brand managers can develop luxury brands with a low visual prominence and discreet signals in their design. In showing how Chinese brand managers turn away from mass-market luxury branding and work on authentic value projects through inconspicuous branding, our results also extend the brand development theory.

Second, we also contribute to luxury brand development research by linking authenticity construction theory with inconspicuous branding. With the rise of inconspicuous consumption (Eckhardt et al., 2015; Wu and Luo, 2017), brands can leverage the trend to develop luxury brands. Building upon models of authenticity construction, our finding illustrates a new way of brand development through quality commitment (the use of nature places and rare raw materials), heritage (the use of traditional and historical elements and symbols), and sincerity (the use of sincere story and sustainability). This finding provides more details of craft brand authenticity, although Napoli et al. (2016) provide the three dimensions of brand authenticity roughly.

## Practical Implication

Our findings provide managerial implications to brand managers. Kapferer (2015) suggests the conspicuous consumption in China. But Wu and Luo (2017) indicate the rise of inconspicuous consumption with four types. Followed the research of Wu and Luo (2017), this paper demonstrated that in China, an inconspicuous approach to branding is on the rise. Inconspicuous brand development methods in China may assume various forms, given cultural, historical, and political contexts. Our findings suggest that an inconspicuous approach to Chinese luxury brands is derived from the rise of inconspicuous consumption in China and a rejection of becoming status brands due to being less famous than well-known Western brands, superficiality of status branding, and limited production capability. In this sense, inconspicuous Chinese branding may signal not a departure from the deeper aspects and practices of the global marketing field but rather the greater acculturation, appropriation, and differentiation of global luxury marketers.

Beverland (2005) observes that brand managers typically use the production technique, history, tradition, and place to craft brand authenticity in the wine industry. His research further shows how wine brand managers craft authenticity to develop status brands. However, our results suggest that inconspicuous branding is not simply catering inconspicuous consumption without well thought branding. Luxury brand managers need to craft authenticity in relation to inconspicuous branding. Authenticity is reflected in relating the brand to a particular place, and in our results, it is relating to nature, culture, and sustainability. Moreover, this research employs ethnographic study to provide more cultural flows and psychological motives of inconspicuous branding through crafting brand authenticity, which provides brand managers approach to conduct brand development study.

## Limitation and Further Studies

This paper examines why and how Chinese brand managers craft brand authenticity to develop inconspicuous brands. More studies in the future examine the ways of inconspicuous branding and authenticity from historical perspective. Furthermore, this paper employs ethnographic study to explore how Chinese brand managers build inconspicuous brands through crafting brand authenticity. But this paper does not provide experimental data to verify influencing factor of inconspicuous branding and brand authenticity. In the future, we adopt experiment study to examine what factors influence inconspicuous branding and brand authenticity. Moreover, although ethnographic study requires more than 10 interviews or other data, this paper conducted 21 interviewers within 10 Chinese luxury brands, which is mentioned by interviewers. In the further, we could collect more date from field studies which involves more Chinese luxury brands.

## Data Availability Statement

The original contributions presented in the study are included in the article/supplementary material, and further inquiries can be directed to the corresponding author.

## Author Contributions

The author confirms being the sole contributor of this work and has approved it for publication.

## Conflict of Interest

The author declares that the research was conducted in the absence of any commercial or financial relationships that could be construed as a potential conflict of interest.

## Publisher’s Note

All claims expressed in this article are solely those of the authors and do not necessarily represent those of their affiliated organizations, or those of the publisher, the editors and the reviewers. Any product that may be evaluated in this article, or claim that may be made by its manufacturer, is not guaranteed or endorsed by the publisher.
